# The Neutralizing Face of Hepatitis C Virus E2 Envelope Glycoprotein

**DOI:** 10.3389/fimmu.2018.01315

**Published:** 2018-06-11

**Authors:** Netanel Tzarum, Ian A. Wilson, Mansun Law

**Affiliations:** ^1^Department of Integrative Structural and Computational Biology, The Scripps Research Institute, La Jolla, CA, United States; ^2^Skaggs Institute for Chemical Biology, The Scripps Research Institute, La Jolla, CA, United States; ^3^Department of Immunology and Microbiology, The Scripps Research Institute, La Jolla, CA, United States

**Keywords:** hepatitis C virus, neutralizing antibodies, crystal structure, neutralizing face, vaccine design

## Abstract

The high genetic variability of hepatitis C virus, together with the high level of glycosylation on the viral envelope proteins shielding potential neutralizing epitopes, pose a difficult challenge for vaccine development. An effective hepatitis C virus (HCV) vaccine must target conserved epitopes and the HCV E2 glycoprotein is the main target for such neutralizing antibodies (NAbs). Recent structural investigations highlight the presence of a highly conserved and accessible surface on E2 that is devoid of N-linked glycans and known as the E2 neutralizing face. This face is defined as a hydrophobic surface comprising the front layer (FL) and the CD81 binding loop (CD81bl) that overlap with the CD81 receptor binding site on E2. The neutralizing face consists of highly conserved residues for recognition by cross-NAbs, yet it appears to be high conformationally flexible, thereby presenting a moving target for NAbs. Three main overlapping neutralizing sites have been identified in the neutralizing face: antigenic site 412 (AS412), antigenic site 434 (AS434), and antigenic region 3 (AR3). Here, we review the structural analyses of these neutralizing sites, either as recombinant E2 or epitope-derived linear peptides in complex with bNAbs, to understand the functional and preferred conformations for neutralization, and for viral escape. Collectively, these studies provide a foundation and molecular templates to facilitate structure-based approaches for HCV vaccine development.

Hepatitis C is a worldwide epidemic that can cause liver failure and hepatocellular carcinoma. Hepatitis C virus (HCV) infects 1–2% of the world population with estimated 1.5–2 million new infections each year (1–4). Direct-acting antivirals have now been developed to treat patients with persistent HCV infection, yet the reports of increasing number of new HCV infections highlight the urgency in developing an effective HCV vaccine for global control of HCV infection (5).

Hepatitis C virus is an enveloped, positive-strand, RNA virus classified within the *Hepacivirus* genus, one of the four genera of the *Flaviviridae* virus family. The HCV particles consist of a nucleocapsid containing the viral genome surrounded by an endoplasmic reticulum-derived membrane crowned by the E1–E2 envelope proteins (6). It was suggested that the HCV particle is a hybrid lipoviral particle (7) that incorporates a thick shell of host-derived apolipoproteins coating the viral surface (8) and may reduce virus sensitivity to neutralizing antibodies (NAbs) (9, 10). This unique coating of the HCV virion is structurally distinct from other members of the *Flaviviridae* family. The E1 and E2 are type I transmembrane glycoproteins with C-terminal transmembrane domains that form a heterodimer on the viral envelope to enable viral entry into the host cells (11). Of note, it has been shown in mammalian cell expression systems that E1 and E2 form noncovalent heterodimers (12, 13), whereas in the cell culture HCV system, the virion-associated E1–E2 complex can be linked covalently by disulfide bonds (14). It is unclear that which form represents the functional E1E2 heterodimer, or whether they could represent different maturation stages of E1E2.

Hepatitis C virus entry is a complex and multistep process that involves interactions of the viral particles with cell surface glycosaminoglycans and many host factors, with the tetraspanin CD81, scavenger receptor class B member 1 (SR-B1), claudin-1, and occludin considered to be the essential set of entry factors (15–18). E2 may serve as the receptor binding protein of HCV and directly interacts with the CD81 and the SR-B1 [for review see Feneant et al. (19)]. In contrast, the role of E1 is poorly understood and appears to help modulate the E2-receptor interactions and fusion with the host cell membrane (20–22).

E2 is the main target of NAbs and it has been suggested that the major mechanism for HCV neutralization is blockage of interaction between E2 and its receptor CD81 (23). Several broadly NAbs (bNAbs) have been isolated from infected patients or immunized animals. The majority of these bNAbs target three overlapping/adjacent neutralizing sites (as defined by antibody competition) and block E2 binding to the CD81 receptor [for review see Ref. (23, 24)]. These epitopes include antigenic site 412–423 (AS412, antigenic domain E, or epitope I), antigenic site 434–446 [AS434, part of E2 front layer (FL), antigenic domain D, or epitope II], and antigenic region 3 (AR3). When the first E2 structure was determined, these neutralizing sites were found to cluster on an exposed surface devoid of glycans on E2, known as the neutralizing face (25). Here, we summarize recent knowledge on the E2 neutralizing face, based on structures of the E2 core domain and peptide–bNAb complexes corresponding to different E2 epitopes.

## Structural Studies of E2 Envelope Glycoprotein

Structural studies of the HCV envelope glycoproteins are essential for a better understanding of the viral entry mechanism as well as for vaccine and drug design. Yet, since overexpression of the HCV envelope glycoproteins often results in misfolded or aggregated proteins, structural studies have been technically challenging. To date, there are no available high-resolution structures of the E1E2 heterodimer, the entire E1, or the entire E2. Moreover, since the E2 transmembrane region is required for folding of E1 (13), only the ectodomain of the E2 can be expressed as a folded and soluble protein (26–28) and, therefore, is more amenable for structural studies.

## E2 Core Domain Structures

**Figure 1:**
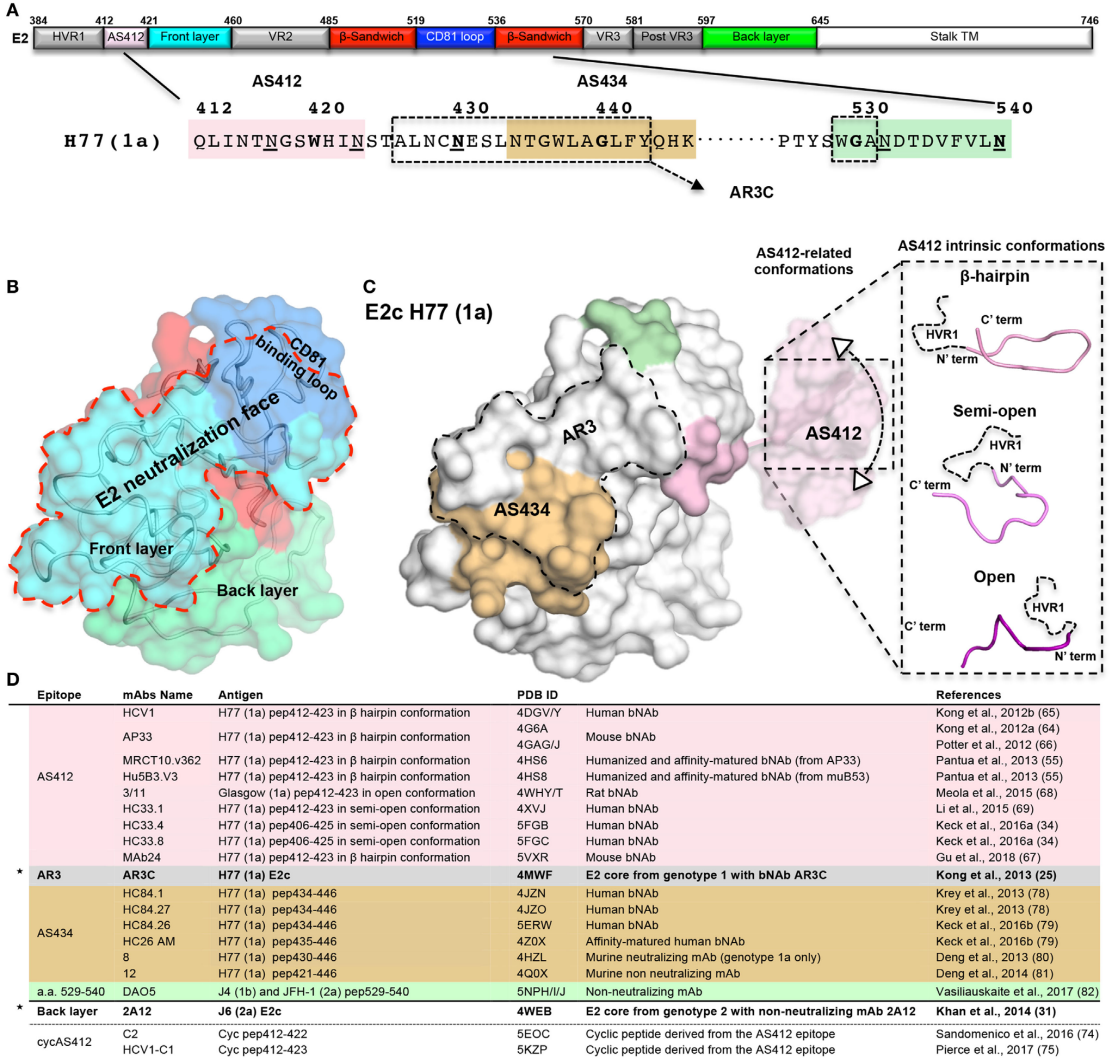
The neutralizing face of the hepatitis C virus E2 glycoprotein. **(A)** Schematic representations of E2 regions (a.a. 384–746, based on the prototypic isolate H77 numbering system) colored by structural components with variable regions in gray, AS412 region in pink, front layer (FL) in cyan, β-sandwich in red, CD81 binding loop (CD81bl) in blue, back layer (BL) in green, and the stalk and transmembrane region (TM) in white. The a.a. sequence of the neutralization face (a.a. 412–446 and 525–535) and the epitope of mAb DAO5 is shown below. The AS412, antigenic region 3, and AS434 neutralization epitopes are marked in pink, dashed rectangle, and wheat. The epitope of the non-neutralizing mAb DAO5 is marked in green. The N-linked glycosylation sites surrounding the neutralizing face (N417, N423, N430, N532, and N540) are underlined. **(B)** Surface representation of the E2c structure (25) (PDB entry 4MWF) with the structural components colored as in **(A)**. The neutralizing face is marked by a red dashed line. **(C)** The E2 neutralizing epitopes on the E2c structure. For the AS412 epitopes, a.a. 412–420 are modeled onto E2c based on the AP33 bNAb-AS412 crystal structure (PDB entry 4G6A). The conformational flexibility of AS412 related to the E2 (30) is schematically shown. The three known AS412 conformations (β-hairpin, semi-open, and open) for neutralization are shown on the right. **(D)** Summary of the E2 crystal structures. The two E2 core domain-mAb structures are marked by stars.

The E2 glycoprotein (amino acid 384–746 in the H77 prototypic strain) is heavily modified post translationally by up to 11 N-linked glycans (29) and 9 strictly conserved disulfide bonds. E2 possesses three variable regions (VRs), hypervariable region 1 (HVR1), and VRs 2 and 3 (VR2 and VR3, Figure 1A), that comprise ~25% of the E2 sequence and contribute to the high genetic diversity of HCV. The VRs and N-linked glycans increase the inherent heterogeneity of E2, which in turn influence the accessibility of antibody epitopes. The E2 ectodomain is a highly stable protein with a melting temperature (T_m_) of ~85°C (30). Yet, two independent hydrogen–deuterium exchange (HDX) mass spectrometry experiments indicate high flexibility of the E2 protein, mostly in the VRs, the FL, and CD81 binding loop (CD81bl) (30, 31) that further hinder structural studies of E2.

To determine the structure of E2, the E2 ectodomain was engineered by removal of the E2 flexible regions in two independent studies (25, 31). In both cases, a bound mAb facilitated crystallization of E2 (Figures 1B,D). The first structure of the prototypic strain H77 isolate (genotype 1a) in complex with bNAb AR3C (see below), consists of E2 residues 412–645 with an internal truncation of VR2 and removal of the N448 and N576 glycosylation sites (E2c) (25). The second structure, of the J6 isolate (genotype 2a) in complex with non-neutralizing mAb 2A12 that binds to the back layer (BL), consists of E2 residues 456–656 (456–652 based on H77 isolate numbering) (31). Overall, both structures share a similar fold but with significant conformational variation around the VR3 region (564–612) and some differences in their disulfide bonds (32). The E2 core domain adopts a globular structure with a new protein fold consisting of a central immunoglobulin (Ig) β-sandwich fold that is stabilized by conserved disulfide bonds and flanked by a FL and a BL (N- and C-terminally). FL is mostly a β-strand with a short helix that packs against the central β-sandwich and BL consisting of antiparallel β-sheets and short helices (Figures 1A,B). Both E2 structures indicate that more than 60% of the residues are disordered or in loops, despite the Ig β-sandwich scaffold being highly stabilized by disulfide bonds that can accommodate conformational flexibility of VRs and FL (30).

## The E2 Neutralizing Face

Based on the H77 E2c structure and epitope mapping experiments, four structural surface regions, or faces, are defined: glycan face, occluded face, non-neutralizing face, and neutralizing face (25). Of note, FL and CD81bl are not modeled in the J6 E2 structure (31). The neutralizing face is a predominantly hydrophobic surface that overlaps most of FL (421–459) and CD81bl (519–535, Figures 1A,B) (25) and consists of highly conserved residues. The neutralizing face is accessible on the viral surface and is immunogenic both in infection and in immunization (23, 33). Negative-stain electron microscopy (EM) of the E2 ectodomain in complex with bNAb AR3C suggested that, although surrounded by N-glycosylation sites, the neutralization face is not obstructed by glycans (excluding the AS412 region, see below) or VRs. Moreover, the neutralization face can be recognized by NAbs with different angles of approach to E2 (30). Intriguingly, it was recently suggested that non-neutralizing mAbs that target HVR1 (34) could shield the neutralizing face and protect HCV from binding of NAbs.

## E2 Antigenic Region 3

The AR3 is a cluster of discontinuous epitopes formed by E2 FL and CD81bl (Figure 1A) that was originally defined by a panel of human antibodies isolated from a chronically infected HCV patient (35, 36). The AR3 is a target for bNAbs AR3A, AR3B, AR3C, and AR3D that exhibit cross-genotype neutralization by blocking E1E2 binding to CD81. The AR3 mAbs have been demonstrated to protect against HCV in passive antibody transfer experiments in both the human hepatocyte-chimeric mouse model and the genetically humanized mouse model (35, 36). The AR3 mAbs share a similar genetic background with their heavy chain (HC) encoded by the germline gene family *V_H_1–69* (36), which is known to be germline gene precursors for the generation of bNAbs against HCV (37–39), influenza (40–43), and HIV (44). This group of mAbs interacts with conserved hydrophobic residues in their antigens *via* hydrophobic residues at the tip of their complementarity-determining region 2 loops. Recently, two independent studies reported the isolation of bNAbs, from patients spontaneously cleared HCV, also target AR3 and are encoded by *V_H_1–69* genes (45, 46).

Alanine scanning mutagenesis experiments together with the structural analysis of H77 E2c–AR3C complex mapped the AR3 epitopes to the E2 FL (426–443) and the tip of the CD81bl (529–531) (25, 35, 47), overlapping with the majority of E2 neutralizing face (Figures 1B,C). AR3 comprises mostly highly conserved residues across the HCV genotypes (25) although variability has been observed in several binding residues (e.g., E431, L433, and F442).

The structure of the E2c in complex with the AR3C bNAb indicates a well-defined secondary structure of AR3, where E2 FL consists of β-strands and an α-helix (436–443) that packs against the β-sandwich region and BL. However, this defined conformation is probably induced or stabilized by binding of the AR3C mAb. When unbounded, AR3 on recombinant E2 is highly flexible as shown by HDX mass spectrometry and molecular dynamics simulations (30). Such flexibility may explain the poor quality of NAb responses to the E2 neutralizing face in immunization studies using recombinant E2.

## E2 Antigenic Site 412–423 (AS412)

AS412 is a highly conserved linear antigenic site that overlaps with the N-terminal region of E2 neutralizing face and contains residues that are critical to CD81 binding [e.g., W420 (48)]. AS412 (412–423) is located between the C-terminus of HVR1 and the N-terminus of FL and contains the first two N-glycosylation sites (N417 and N423) of E2 (Figure 1A). AS412 is the target for some of the most characterized cross-genotype NAbs, isolated from both infected donors and E2-immunized animals (49–56) (Figure 1D). Moreover, bNAbs against AS412 show passive protection in animal models (chimpanzee and humanized mice) inoculated with HCV (57, 58) as well as delaying HCV recurrence post-transplant in clinical trials [for HCV1 bNAb (59, 60)]. However, natural elicitation of such bNAbs in infection is rare and is detected only in 2–15% of the patients (54, 61, 62). In animal immunization experiments, only low levels of NAbs against AS412 have been elicited (23, 63).

Although AS412 is present in the H77 E2c construct, only its C-terminus (421–423, Figures 1A,C) could be modeled in the E2c–AR3C complex structure, suggesting high flexibility of this region. The flexibility or conformational heterogenity of AS412 relative to E2 was validated by a recent EM study on the H77 E2c-HCV1 bNAbs complex, which revealed a 10–22° variation in the angle that the HCV1 Fab fragment approaches E2 (30) (Figure 1C, left). A second level of flexibility, likely reflecting the intrinsic conformational variability of the region, was observed in crystal structures of linear peptides corresponding to AS412 in complex with different NAbs. Three main conformations have been reported for AS412 in these antibody complexes (Figure 1C, right). The most common and the first to be determined is the β-hairpin conformation, as observed with HCV1, AP33, MRCT10.v362, hu5B3.v3 bNAbs, and MAb24 (55, 64–67) (Figures 1C,D). An extended or “open” conformation was observed in the complex with rat mAb 3/11 (68) and a semi-open conformation in complexes with mAbs HC33.1, HC33.4, and HC33.8 (Figures 1C,D) (34, 69). Despite these differences in the AS412 conformations, alanine scanning mutagenesis and structural analysis indicate that L413, G418, and W420 are critical for binding of AS412 bNAbs [beside 3/11, see below (70)].

### The β-Hairpin Conformation

The first conformation of AS412 to be determined and the most common is the β-hairpin conformation, stabilized by a number of internal backbone hydrogen bonds, with a β-turn at residues 416–419. In complexes of HCV1, AP33, MRCT10.v362, hu5B3.v3 bNAbs, and MAb24, the β-hairpin conformation is highly similar with slight changes in the β-turn type [type IV hairpin turn in the hu5B3.v3 complex, while type I for all of the others (55, 64–67)]. The hydrophobic face of the hairpin is recognized by a binding pocket composed of the antibody heavy and light chains, whereas the N417 and N423 glycosylation sites project from the opposite side of the peptide and are solvent exposed (65), indicating that AS412 is likely not closely packed against E2. Superposition of the AS412 C-terminus (421–423) of AP33 and HCV1 on the E2c structure results in steric clashes between the FL and the epitope-bound mAb, supporting the notion that AS412 is flexible on E2. Escape of HCV from neutralization by bNAbs targeting AS412 has been reported in several studies (55, 57, 59, 67, 71, 72), including the N415D/K and N417S/T mutations. The N417S/T mutations can result in a glycosylation shift from N417 to N415. Structural analysis of these AS412 complexes provides an explanation for the viral escape mechanism. The side chain of N415 is buried in the antibody binding pocket and, therefore, mutation of N415 or the glycosylation shift to N415-glycan would create steric clashes in the antibody binding pocket and interfere with antibody binding.

### The Semi-Open Conformation

The semi-open conformation was observed in the complex structures with human bNAbs HC33.1, HC33.4, and HC33.8 (34, 69). In this conformation, beside residues 414 and 415 that form an antiparallel β-sheet with the long HC CDR3, the antigen adopts an extended conformation that is stabilized by one internal backbone hydrogen bond (69). Residues 416–419 adopt a β-turn conformation as in the original β-hairpin structures (69). The neutralization potency of the HC33 bNAbs is not impaired by the N417S/T mutation and the glycosylation shift to N415 because the side chain of N415 (as well as N417 and N423) is solvent exposed in the antibody–peptide complex structure (54, 69, 73). Modeling of N-linked glycans on N415 indicates potential interactions with the HC33 HC (69) that may explain the higher neutralization potency of HC33.1 against glycan-shifted virus (73). These properties indicate that the semi-open conformation would be a useful template for structure-based vaccine design.

### The Open Conformation

The extended open conformation of the AS412 was observed in its crystal structure with rat NAb 3/11 (68). This conformation is stabilized by internal backbone hydrogen bonds, similarly to the β-hairpin conformation, but through creation of a different interaction network. The side chains of N415, W420, and H421 are critical for the binding of 3/11 (68, 70). AS412 is immersed in a deep cavity formed by both the HC and LC of 3/11 with only the side chains of N417 and S419 exposed to solvent, providing a structural explanation for viral escape by point mutations N415Y and G418D and the glycosylation shift mutation N417S (68, 71, 72).

### Cyclic AS412

So far two groups have reported structure-based design of AS412 as cyclic immunogens (cycAS412) (74, 75) (Figure 1D). In the first study, cycAS412 did not elicit NAbs in immunized mice (74). Structural studies of one of these mAbs, C2, in complex with cycAS412, revealed that cycAS412 retains a β-hairpin conformation but binding to the C2 mAb is mediated by the opposite face of the epitope. Consequently, the structure suggests that cycAS412 failed to mimic the AS412 conformation required for binding of bNAbs leading to the lack of neutralization capability. In the second study, another cyclic AS412 peptide, C1, was studied (75). Mice immunized with this cyclic peptide conjugated to a protein carrier produced better binding and NAb responses than the equivalent linear peptide, albeit neutralization was restricted to the virus from which the peptide was derived. In the same study, AS412 was also grafted onto a hairpin at E2c BL region. The addition of a second copy of AS412 to E2c was not detrimental to the engineered protein (T2) and the antibody response elicited appeared to be similar to the standard, soluble, C-terminally truncated, E2 ectodomain (384–661). It is yet to be determined why the antibody response was still restricted to the autologous virus despite NAbs to AS412 being elicited.

## E2 Antigenic Site 434–446 (AS434)

AS434 is a short hydrophobic 1.5 turn α-helix (helix α-1, 437–442) encircled by a N- and C-terminal extended regions spanning FL residues 434–446. Several NAbs that target AS434 have been isolated from chronically infected HCV donors (39) and from immunized mice (76) (Figure 1). Beside NAbs, AS434 can also elicit non-neutralizing mAbs that were proposed to interfere with NAbs that target AS412 (39, 76, 77). AS434 is highly conserved among HCV genotypes (excluding residues 434 and 444) and escape mutants have not been observed *in vitro* (39), indicating that it is a good target for structure-based vaccine design.

AS434 has been structurally defined by six crystal structures of mAbs in complex with the corresponding linear peptides. Four of them are human bNAbs (HC84.1, HC84.27, HC84.26, and HC84.26AM) (78, 79) and the other two are the weakly and non-neutralizing murine mAbs (12 and 8) (80, 81) (Figure 1D). Although some variations are found in the conformation of the N- and C-terminal regions, residues 437–442 of the different peptides adopt an α-helical conformation that is similar to that observed in E2c FL (25). Notwithstanding, the biological activities of the mAbs vary greatly because of the way they approach their epitopes. When superposed on the E2c crystal structure, the human bNAbs bind to AS434 using an angle of approach that is similar to bNAb AR3C with only minor structural clashes with the E2 protein. In contrast, superposition of the murine mAbs onto the E2c structure results in structural clashes with the central β-sandwich scaffold. These clashes suggest that the murine mAbs bind an opposite face of the epitope (almost 180° rotation of the helix) and, therefore, would require some conformational rearrangement of AS434 upon antibody binding. The transition between the two modes of binding is possibly supported by the high flexibility of E2 FL as indicated by HDX experiments (30).

In the structures of the HC84 human bNAbs, the C-terminal but not the N-terminal loop of the AS434 was modeled. Yet, the interactions are dominated by hydrophobic interactions between the side chains of the α-helical residues L441 and F442 and the antibody CDRH2 hydrophobic tip (39, 78). Similar to the AR3 bNAbs, the HCs of HC84 bNAbs also originate from the *V_H_1-69* family genes. In contrast, in the murine mAb 8 structure, only the N-terminal loop was modeled with a different mode of binding to AS434: hydrophilic interaction with the side chains of E431 and N434 and hydrophobic interaction with the side chains of W437 and L438 that are essential for antibody binding. This different mode of binding requires conformational rearrangement of AS434 on E2 to expose the side chains of W437 and L438 buried in the E2c structure.

With the goal of using bNAbs for HCV immunotherapy, a recent study (79) applied yeast display to affinity mature the human HC84.26 bNAb. The affinity-matured mAb, HC84.26AM, showed improved affinity and neutralization against diverse HCV isolates and the capability to protect humanized mice against challenge with infectious human serum. Structural study of HC84.26AM in complex with the AS434 peptide showed that the conformation of the epitope is similar to that with the wild-type mAb, where mutations in the light chain improved the biological activity of the antibody.

## The CD81 Binding Loop

CD81bl, spanning residues 519–535, connects β-strands 5 and 6 of the E2 β-sandwich scaffold and contains critical residues for CD81 receptor binding, including Y527, W529, G530, and D535 (48). Although fully modeled as a loop in the H77 E2c structure, the CD81bl is highly flexible when unbound as indicated from HDX experiments (30) and disordered in the J6 E2 core structure (31). Intriguingly, the alanine scanning mutagenesis indicates that many residues, despite being distal from the CD81 binding surface, can severely suppress E2 binding to CD81 when mutated (47, 48). These results suggest that the overall conformation of CD81bl is important in positioning W529, G530, and A531 toward E2 FL so as to form the receptor binding site and neutralizing face of E2.

The mAb DAO5 is a non-neutralizing mAb that can compete with CD81 binding to soluble E2 (82), and targets the C-terminus of CD81bl and β-strand 6 of the β-sandwich scaffold region (residues 529–540). Crystal structures of mAb DAO5 in complex with peptides derived from the J4 and JFHI isolates (82) indicate that the peptide adopts a one-turn α-helical conformation with the side chains of F537 and L539 buried in the antibody binding interface. In the E2c–AR3C structure, this region adopts a β-strand conformation with F537 and L539 side chains pointing toward the core of the β-sandwich. Since DAO5 is non-neutralizing, it is possible that it recognizes misfolded E2 presented in the antigens used in immunization.

## Conclusion

The mechanisms used by HCV, as well as by HIV and influenza virus, to evade the humoral immunity include high genetic variability, glycan shielding of immune epitopes, and conformational flexibility near the neutralizing sites on the viral envelope proteins (83). An effective vaccine for HCV must address these challenges, *inter alia*, by targeting conserved neutralizing epitopes to improve the immune response. Despite the challenges in structural studies of HCV envelope glycoproteins, the recent E2 structures (E2c and linear epitopes) have contributed to the identification of the E2 neutralizing face. Structural characterization of HCV antigen–antibody complexes has improved our understanding of how the immune system recognizes HCV to achieve broad neutralization. These studies provided the field with useful molecular templates to enable structure-based design of candidate vaccine antigens.

## Author Contributions

NT, IAW, and ML prepared and wrote the review manuscript.

## Conflict of Interest Statement

The authors declare that the research was conducted in the absence of any commercial or financial relationships that could be construed as a potential conflict of interest. The handling Editor declared a past co-authorship with the authors.
